# The *Helioseismic and Magnetic Imager* (HMI) Vector Magnetic Field Pipeline: Magnetohydrodynamics Simulation Module for the Global Solar Corona

**DOI:** 10.1007/s11207-015-0686-z

**Published:** 2015-04-23

**Authors:** K. Hayashi, J. T. Hoeksema, Y. Liu, M. G. Bobra, X. D. Sun, A. A. Norton

**Affiliations:** W. W. Hansen Experimental Physics Laboratory, Stanford University, Stanford, CA USA; National Astronomical Observatory of China, Chinese Academy of Sciences, Beijing, China

**Keywords:** Instrumentation and data management, Magnetic field, Photosphere, Corona, Interplanetary, Magnetohydrodynamics

## Abstract

Time-dependent three-dimensional magnetohydrodynamics (MHD) simulation modules are implemented at the Joint Science Operation Center (JSOC) of the *Solar Dynamics Observatory* (SDO). The modules regularly produce three-dimensional data of the time-relaxed minimum-energy state of the solar corona using global solar-surface magnetic-field maps created from *Helioseismic and Magnetic Imager* (HMI) full-disk magnetogram data. With the assumption of a polytropic gas with specific-heat ratio of 1.05, three types of simulation products are currently generated: i) simulation data with medium spatial resolution using the definitive calibrated synoptic map of the magnetic field with a cadence of one Carrington rotation, ii) data with low spatial resolution using the definitive version of the synchronic frame format of the magnetic field, with a cadence of one day, and iii) low-resolution data using near-real-time (NRT) synchronic format of the magnetic field on a daily basis. The MHD data available in the JSOC database are three-dimensional, covering heliocentric distances from 1.025 to 4.975 solar radii, and contain all eight MHD variables: the plasma density, temperature, and three components of motion velocity, and three components of the magnetic field. This article describes details of the MHD simulations as well as the production of the input magnetic-field maps, and details of the products available at the JSOC database interface. To assess the merits and limits of the model, we show the simulated data in early 2011 and compare with the actual coronal features observed by the *Atmospheric Imaging Assembly* (AIA) and the near-Earth *in-situ* data.

## Introduction

The Joint Science Operation Center (JSOC) data pipeline currently processes various types of observations made by the *Helioseismic and Magnetic Imager* (HMI) instrument (*e.g.* Scherrer *et al.*, 2012; Schou *et al.*, 2012) and the *Atmospheric Imaging Assembly* (AIA) instrument (*e.g.* Lemen *et al.*, 2012) onboard the *Solar Dynamics Observatory* (SDO). From the HMI observations, the data pipeline rapidly produces various types of magnetic-field data (see Liu *et al.*, 2012; Hoeksema *et al.*, 2014; Bobra *et al.*, 2014; Centeno *et al.*, 2014) as well as helioseismology data and derivative products (Zhao *et al.*, 2012). Details of the data production processes for the line-of-sight magnetograms and vector magnetic field are described by Liu *et al.* (2012) and Hoeksema *et al.* (2014), respectively.

HMI observes the full-disk Sun continuously and almost seamlessly in time, with one-arcsecond spatial resolution and rapid temporal cadence, which allows us to generate frequent whole-Sun maps, such as the synoptic map, representing the solar-surface conditions on a time scale of one solar rotation with fine spatial scale.

The HMI observables do not include the solar corona (above the photosphere), which is not directly measured. For various purposes, specifically for space weather, the theoretically determined three-dimensional solar corona and solar wind that depend on the magnetic field measured in the solar photosphere are crucially important.

As demonstrated by pioneering works, (*e.g.* Wu, Dryer, and Han, 1983; Linker, van Hoven, and Schnack, 1990; Dryer *et al.*, 1991; Washimi and Sakurai, 1993; Usmanov, 1993), a time-dependent magnetohydrodynamics (MHD) simulation can provide a powerful capability to inspect the complex nonlinear state of the global trans-Alfvénic solar corona and solar wind. Specifically, by using the global solar-surface magnetic-field map as an input to specify the boundary values, the MHD solution can represent the state of the solar corona at the time of observation (*e.g.* Usmanov, 1993; Usmanov and Dryer, 1995). Using time-relaxation MHD simulations, the simulated MHD variables interact with each other until the system reaches the minimum-energy state of the sub- and trans-Alfvénic solar corona and super-Alfvénic solar wind. This solution can be regarded as the most plausible quiescent state of the global solar atmosphere. The large-scale steady state is a cradle within which the magnetic energy in the streamer or small-scale active regions evolves and accumulates, and it defines a background state where interplanetary disturbances propagate. As such, it is beneficial to the solar-physics community to have available MHD data products generated regularly at the JSOC data center.

Various MHD models (*e.g.* Suess, Wang, and Wu, 1995; Mikic *et al.*, 1999; Usmanov *et al.*, 2000; Nakamizo *et al.*, 2009; Feng *et al.*, 2010; Usmanov *et al.*, 2011) have applied sophisticated models of coronal heating and acceleration, such as Alfvén wave decay and the radiation process, to successfully produce realistic features of the solar corona and solar wind, such as steep radial gradients (or acceleration) of the solar-wind speed in the solar corona, final velocities in distant regions, and temperatures in coronal streamers, active regions, and coronal holes. In general, these models are associated with the heat conduction term that has a much shorter time scale than the global Alfvén waves; thus it requires intensive computation to compute them. Unfortunately, as one of the modules at the HMI data pipeline, it is not practical for us to implement such computationally expensive models.

The spatial resolution of the MHD simulation is another factor that we have to consider in estimating the computational resources needed. In the spherical coordinate system, a straightforwardly constructed grid system will have a longitudinal grid size of *r*Δ*ϕ*sin(*δθ*)≈*r*Δ*ϕ*Δ*θ* near the polar axis, which is approximately proportional to (Δ*ϕ*)^3^ if Δ*ϕ*≈Δ*θ*. Roughly, the time step is inversely proportional to the smallest size of the grid, and the spatially high-resolution simulation cannot be regularly conducted with limited computational resources.

In practice, low-resolution simulations generated in a timely manner will be more beneficial as the standard derivative product in the HMI data pipeline. For example, quick-look simulation data will help us grasp the current state of the solar corona and solar wind, and a sudden change found in quick-look data offers early warnings to recognize at least the possibility of global-scale variations worth further examination.

Since early 2008, well before the HMI observations began in May 2010, we started running daily MHD simulations using the *Solar and Heliospheric Observatory* (SOHO)/*Michelson Doppler Imager* (MDI) data. Through preparation runs using our existing MHD code (Hayashi, 2005), several choices had been tested. Simulation plots can be found at hmi.stanford.edu/MHD/daily_mhd.html. Several attributes are necessary for data products in the HMI pipeline, such as timeliness of production of near-real-time (NRT) data. Computational resources are another factor that we have to take into account. Based upon the experience gained through the test runs, we choose to run low-resolution (≈ 5.6 degrees) MHD simulations on a daily, NRT basis, and medium-resolution (≈ 2.8 degrees) simulation once each Carrington rotation, with a set of simulation settings that have been optimized to provide the optimal combination of the MHD simulations and HMI observations to the solar-physics community as an HMI data product. The MHD data as well as source codes for this module can be found at jsoc.stanford.edu.

This article describes the details of the magnetic-field map production process, the MHD model settings, and the new data products available at the JSOC. This article is organized as follows: In Section 2 we describe two types of input magnetic-field maps, the synoptic maps and the synchronic maps, regularly generated at the JSOC. In Section 3, details of the MHD models employed are given. In Section 4, information needed to access and specify the MHD data using the JSOC data interface is provided. Section 5 demonstrates the results of MHD simulations and makes comparisons with the AIA coronal images and the near-Earth *in-situ* OMNIweb data set over a selected period from late 2010 to early 2011. A summary and remarks are given in Section 6

## Synoptic and Synchronic Maps of the Magnetogram Data

Synoptic maps of the magnetic field have been widely used for models of the global corona, such as the potential-field source-surface (PFSS) (Schatten, Wilcox, and Ness, 1969; Altschuler and Newkirk, 1969) and MHD simulation models. We can specify realistic conditions of the magnetic field at a time of interest for the coronal models by using the whole-Sun map of the observed solar photospheric magnetic field data in a synoptic map or a synchronic frame.

When making full-surface maps on a daily, NRT basis, we need to implement some extra procedures, such as reducing the distortion of the map at high latitudes due to the differences between the differential solar rotation and the assumed rigid rotation. We have developed an improved whole-Sun map, called the synchronic frame (*e.g.* Zhao, Hoeksema, and Scherrer, 1999), and we use it to create the daily updated maps in the HMI data pipeline.

### The Synoptic Chart and Synchronic Frame in JSOC

**Figure 1:**
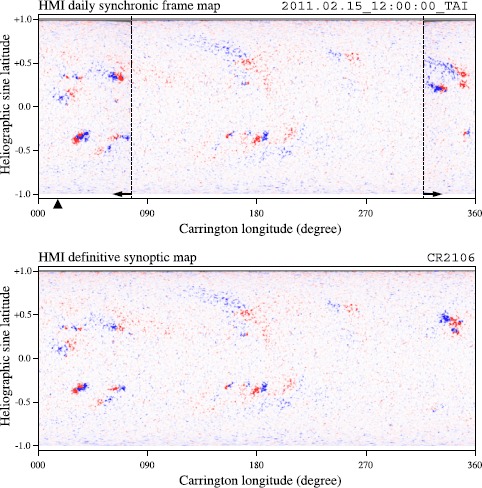
The synchronic frame for 15 February 2011 (top) and synoptic map for CR 2106, from 20 January 2011 to 16 February (bottom). In the top panel, two dotted vertical lines are placed at the bounding longitudes of the updated part. Two arrows indicate the 120-degree updated region, and a triangle is placed at about 16 degrees Carrington longitude that corresponds to the central meridian on 15 February 2011, 12:00 UT. Blue (red) represents the positive (negative) polarity. The colors are truncated at ± 100 Gauss.

Figure 1 shows an example of the once-per-CR standard synoptic map and daily synchronic map of *B*_*r*_ that the MHD modules use.

In the HMI data pipeline, a standard Carrington synoptic chart is made as follows: First, the line-of-sight field is converted to the radial component by dividing by the cosine of the center-to-limb angle (Wang and Sheeley, 1992). The conversion to the radial-field component means that the B-zero angle [*B*_0_] has been accounted for, and no additional correction for the dependence on *B*_0_ and other projection effects is required. The radial component is then remapped to a Carrington coordinate grid. Each point in the grid has been adjusted to the time of its central meridian to minimize additional smearing due to the differential rotation, as suggested by Ulrich *et al.* (2002). The remapped grid retains HMI’s spatial resolution at the disk center, *i.e.* 0.03 degrees. It is then reduced to 0.1 degrees by convolving with a two-dimensional Gaussian with a half width of three pixels. The field strength is then averaged from all of the contributing remapped magnetograms. Currently, the average is done from twenty 720-second magnetograms that contribute pixels observed around the central meridian. Therefore, the effective temporal width of the HMI synoptic map is about four hours at each Carrington longitude, *i.e.* within two hours of central meridian passage, or within about 1.2 degrees of longitude. The line-of-sight field map is derived from the radial-field chart by multiplying by the cosine of the latitude for each grid pixel. Therefore, the data are Equator-centered, meridian-centered, line-of-sight field values as if observed from a point on the solar Equator.

A daily updated synchronic frame radial field is produced from the remapped HMI 720-second radial-field magnetograms and the standard Carrington synoptic map of the radial field: the remapped magnetograms make up a 120-degree daily updated region between the two edges at 60 degrees of longitude from the central meridian at 1200 UT. A synoptic chart makes up the rest of the daily updated synchronic map. The data values in the daily updated part are four-hour averages of the remapped magnetograms. This four-hour averaging, using twenty 720-second magnetograms, ensures that noise in the updated part matches that of the synoptic chart. There are two types of daily updated synoptic maps. One is from NRT 720-second line-of-sight magnetograms. The other is from the definitive 720-second line-of-sight magnetograms generated with a delay of about 45 days. The size of the maps is 3600 by 1440 with longitude in *x*-axis and sin(latitude) in *y*-axis. Smaller size synoptic maps (720×360) are also produced.

Table 1 tabulates the series names, the topmost identifier in the JSOC database, for the standard synoptic map and daily updated synchronic frames. The MHD modules use the radial-component maps (marked with asterisks (*) in Table 1). As soon as an NRT synchronic frame for 1200 UT is generated, a low-resolution NRT MHD run starts, and the MHD data become available around 22 UT at the latest. The definitive version of the daily map and the standard once-per-CR synoptic map are usually generated 45 days after the last observation, when all necessary calibration processes are completed.

**Table 1:** Series names of synoptic and synchronic maps.

Series name	Description
hmi.Synoptic_Mr_720s*	Radial-component standard synoptic map
hmi.Synoptic_Ml_720s	Line-of-sight component standard synoptic map
hmi.Mrdailysynframe_720s*	Radial-component daily updated map
hmi.Mldailysynframe_720s	Line-of-sight component daily updated map
hmi.Mrdailysynframe_720s_nrt*	Radial-component NRT daily updated map
hmi.Mldailysynframe_720s_nrt	Line-of-sight component NRT daily updated map

## MHD Model of the Global Corona

The main part of the three-dimensional time-dependent MHD simulation code is almost identical to the one used in our previous works (*i.e.* Hayashi, 2005; Hayashi, Zhao, and Liu, 2008, and Hayashi, 2013). In brief, it employs fairly standard concepts in time-dependent MHD simulation, such as the Total Variation Diminishing (TVD) (*e.g.* Brio and Wu, 1988; Roe and Balsara, 1996) and Monotonic Upstream Scheme for Conservation Laws (MUSCL) (*e.g.* van Leer, 1979). One notable feature in our code is that the concept of the projected normal characteristics method (Nakagawa, Hu, and Wu, 1987; Wu and Wang, 1987; Han, Wu, and Dryer, 1988) is applied to treat the solar-surface inner boundary sphere. This method can provide a physically consistent environment without artifacts of incoming waves from outside the simulated volume, keeping the sinusoidality of the magnetic field (*e.g.* Yeh and Dryer, 1985). The details of the model are given by Hayashi (2005) and references therein.

To speed up the simulation, we employ the Lax–Friedrichs method instead of the linearized Riemann solver, which contains computationally expensive operations of eight-by-eight left and right eigenvector matrices of MHD hyperbolic equation system. We note that because the concept of the normal projected characteristic method is applied, the same eight-by-eight eigenmatrices are still used in this simplified version.

### MHD Model

Instead of using a coronal heating and acceleration model, we simply assume a near-isothermal polytropic gas with specific-heat ratio [*γ*] of 1.05 to make the trans-Alfvénic solar wind. From provisional tests with various specific-heat ratios from 1.001 to 1.2, we found that 1.05 is best because the ratio can create a moderate contrast in coronal density and plasma speed.

It is easily expected that the polytrope model may result in higher solar-wind speed near the heliospheric current sheet (HCS) originating from the slow-speed region just above the closed-field coronal streamer where higher density is always associated with higher temperature in the modeled solar wind. In addition, the contrast of the simulated solar-wind speed at 1 AU is generally very faint. Thus, we have to be careful when using the data at 1 AU, although this does not mean that generality in the nonlinear MHD interaction processes in the sub/trans-Alfvénic solar corona had been lost. Because the near-isothermal polytrope model has been a standard reference in solar-wind studies probably since the pioneering work of Parker (1958), it is reasonable to use this model even with the caveats for the distant, 1 AU region. Overall, global coronal magnetic-field structures are not sensitive to the heating and acceleration processes, but we still need to be cautious. For example, one of the coronal features that might be sensitive to the choice of the coronal heating is the unipolar boundary layer (UBL) in interplanetary space (Zhao and Webb, 2003) or pseudo-streamers in the corona, below which two compact closed-field structures are located between two coronal holes of the same magnetic polarity. In this configuration, the pressure balance between the magnetic field and plasma might be a critical factor determining whether the simulated system will have UBL or two small closed-field regions with a narrow coronal hole in between.

With the assumption of the near-isothermal polytrope, the basic equations to be solved are the time-dependent MHD equations in the frame rotating at the sidereal angular velocity of solar rotation [**Ω**];

1 $$\begin{aligned} \frac{\partial\varrho}{\partial t} =& -\nabla\cdot(\varrho\boldsymbol{V}) \end{aligned}$$

2 $$\begin{aligned} \frac{\partial(\varrho\boldsymbol{V})}{\partial t} =& -\nabla\cdot \biggl( P_g +\varrho\, \boldsymbol{V}:\boldsymbol{V} -\frac{1}{4\pi}\boldsymbol{B}:\boldsymbol{B}+ \frac{B^2}{8\pi} \biggr) \\ &+\varrho\, \bigl[ \boldsymbol{g} +(\boldsymbol{\Omega}\times\boldsymbol{r}) \times\boldsymbol {\Omega} +2\,\boldsymbol{V}\times\boldsymbol{\Omega} \bigr] -(\nabla\cdot\boldsymbol{B})\boldsymbol{B} \end{aligned}$$

3 $$\begin{aligned} \frac{\partial\boldsymbol{B}}{\partial t} =& -\nabla\cdot(\boldsymbol{V}:\boldsymbol{B}-\boldsymbol {B}:\boldsymbol{V}) -(\nabla\cdot\boldsymbol{B})\boldsymbol{V} \end{aligned}$$

and

4 $$\begin{aligned} \frac{\partial{\mathcal{E}}}{\partial t} =& -\nabla\cdot \biggl( \mathcal{E}+P_g- \frac{1}{4\pi}(\boldsymbol{V}\times\boldsymbol {B})\times\boldsymbol{B} \biggr) \\ &{}+ \varrho\,\boldsymbol{V}\cdot \bigl( \boldsymbol{g}+(\boldsymbol{\Omega} \times\boldsymbol{r})\times \boldsymbol{\Omega} \bigr) -(\nabla\cdot \boldsymbol{B}) (\boldsymbol{V}\cdot\boldsymbol{B}), \end{aligned}$$

where *ϱ*, ***V***, ***B***, *P*_*g*_, $\mathcal{E}$, ***r***, *t*, ***g*** and *γ* are mass density, velocity of plasma flow viewed in the frame rotating with the angular velocity **Ω**=2*π*/25.3 radian day^−1^ (or 14.2 degrees day^−1^), magnetic-field vector, gas pressure, energy density [$\mathcal{E}=\varrho v^{2}/2+P_{g}/(\gamma-1)+B^{2}/2$], position vector originating at the center of the Sun, time, solar gravitational force [$\boldsymbol{g}=-G\mathrm{M}_{\odot}\hat{\boldsymbol{r}}/r^{2}$], and specific-heat ratio, respectively. In this notation, “:” expresses the dyadic tensor product of two vectors. The specific-heat ratio, [*γ*=1.05], is constant everywhere.

The normalizing factors (or typical values) of density, temperature, flow speed, and magnetic field are set as *n*_0_=9.0641×10^4^ count cm^−3^, *T*_0_=1×10^6^ K, *V*_0_=131.66 km s^−1^, and *B*_0_=1.817×10^−2^ Gauss, respectively. Table 2 tabulates the setting of parameters used in this model.

**Table 2:** Parameters in MHD simulation.

Notation	Value	
*γ*	1.05	Specific-heat ratio
*n*_b_	2×10^8^ count cm^−3^	Density at bottom (at 1.01 R_⊙_)
*T*_c_	1 M K	Temperature at critical point (at ≈ 5.5 R_⊙_)
Ω	360 degree/25.3 days	Sidereal solar rotation rate

The initial values of plasma quantities are set as the Parker solution, with the velocity and temperature equal to the normalizing factors at its critical point (at *r*=3.8282×10^6^ km or 5.47 solar radii). The typical density is selected such that the density in the bottom boundary sphere [*n*_b_] will be 2×10^8^ cm^−3^. The normalization factor of the magnetic field is chosen such that the typical magnetic pressure is equal to the typical ram pressure [$B_{0}^{2}/8\pi= n_{0}V_{0}^{2}/2$]. The typical thermal pressure of perfectly ionized hydrogen gas [*P*_0_] is equal to 2*n*_0_*k*_B_*T*_0_, where *k*_B_ is the Boltzmann constant. The initial magnetic field is the solution of the PFSS model with the boundary map given from the upstream modules in the HMI data pipeline.

Because of the cell-centered grid system in our model, the divergence of the magnetic field will inevitably differ from zero. To address this problem, the terms associated with the divergence of the magnetic field [∇⋅***B***] are added to the right-hand side of Equations (2), (3), and (4) so that the effect of the numerical monopole will be minimized and the numerical magnetic monopole will move with the plasma material and finally be swept out of the computational domain (Brackbill and Barnes, 1980; Powell, 1994; Powell *et al.*, 1999; Toth, 2000).

**Figure 2:**
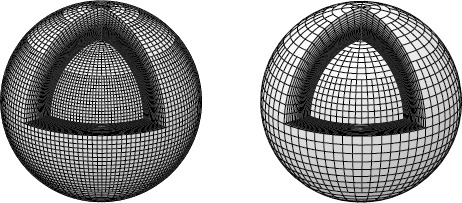
Grid systems of the simulation. (left) The grid system of moderate resolution that is used for definitive once-per-CR run. (right) the lower-resolution grid system. For visibility, only the inner 25 of 72 spherical layers in the radial direction are drawn. The finite-volume method (FVM) merges the cells near the Pole and uses appropriately averaged values. This averaging mitigates the CFL constraint and helps speed up the calculation while preserving the conservative quantities such as mass and total energy.

The grid system is constructed in a spherical coordinate system so that the input whole-Sun map can be hosted straightforwardly. To discretize the space, we employ the concept of the finite-volume method (FVM) (Tanaka, 1995). From our provisional tests, we set the simulated space to be from 1.01 R_⊙_ to 50 R_⊙_ in the radial direction, and from 0 to *π* in the latitudinal direction. This range of heliocentric distance is divided into 72 grid cells (excluding the ghost cells). The innermost five and outermost five grids have uniform radial grid sizes. In the simulation, the latitude runs 180 degrees from North to South (co-latitude) and the longitude runs from West to East, in an equi-angle grid system. The numbers of grids in the latitudinal and longitudinal directions are 32 and 64 in the low-resolution daily simulation and 64 and 128 in the medium-resolution once-per-CR run. If we straightforwardly apply the equi-angle grid system, the sizes of cells near the Poles are small and the time step [Δ*t*] will be problematically small. To mitigate the computational cost due to the severe Courant–Friedrichs–Lewy (CFL) condition, numerical cells near the Poles are merged: as in Hayashi, Zhao, and Liu (2008), the number of cells to be longitudinally merged is calculated to be *N*th power of 2 [2^*N*^], with *N* being the smallest integer satisfying

5 $$ 2^N(r\Delta\phi\sin\theta)\ge\min({\Delta}r,r\Delta\theta) $$

at each latitude [*θ*]. Figure 2 shows the grid systems used in our coronal MHD model.

### Three Simulation Settings

**Figure 3:**
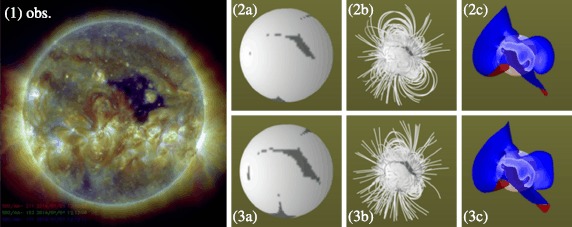
An example of the simulation results and comparison with the AIA measurement. Frame 1 is the three-color composite of AIA images taken around 1200 UT, 1 January 2014. Frames 2a to 2c show the base of the open-field region, coronal field lines, and the contour surface of the zero value of the radial component of the initial PFSS magnetic field. Frames 3a to 3c show the same properties as Frames 2a to 2c, except that the time-relaxed MHD solution is used. The magnetic-field map given to the initial PFSS and MHD model is for CR 2145, whose central time is approximately the date of the AIA observation. The viewpoint in Frames 2a to 3c is set at 182.83 degrees Carrington longitude and 3.2 degrees South heliographic latitude, the approximate position of Earth at the time of the AIA observations.

Considering various factors, such as required computational resources and timeliness of data delivery, we select three types of MHD simulation: (A) A medium-resolution simulation (with 72, 64, and 128 grid points for the radial, latitudinal and longitudinal directions, respectively) that is computed about once every four weeks (once-per-CR). This choice uses a Gaussian-smoothed definitive synoptic map. The initial potential field is calculated with the iterative Laplace solver so that the input map can be identical to the smoothed map. (B) A low-resolution simulation (with 72, 32, and 64 grid points for the three directions) using the definitive daily updated map. The fifth-order spherical harmonic polynomials are used to calculate the initial and boundary magnetic field. (C) A low-resolution simulation with the same setting as (B) except that this choice uses the near-real-time version of the input magnetic field instead of the definitive one. Figure 3 shows the field lines and current sheet in the corona obtained with a medium-resolution simulation (A) using a definitive map for CR 2145, as an example.

The three types of simulation use an identical code except for the grid size. Each run simulates a 40-hour time-relaxation process starting from the initial PFSS and Parker solution. In the current JSOC cluster computer system, the inter-node MPI job capability is disabled to optimize the system for processing a large number of single-CPU jobs. Therefore, the number of CPU cores available to one particular parallel computation is limited to eight. On an 8-CPU node, the efficiency of our MHD code parallelized with OpenMP is almost 800 %, and it takes about three hours to complete a low-resolution daily run and about one day to complete the once-per-CR medium-resolution simulation.

The first and second simulation runs start after the calibration of the full-disk magnetograms is completed and the definitive version of the whole-Sun maps have been created, which usually takes place a month after the observation. The last simulation (C) starts immediately after the daily NRT synchronic map is generated, usually around 14 UT, and the simulated data can be available around 17 UT. The time lag of about five hours between the last full-disk magnetogram observation and the simulation data production is acceptable because the lag is much shorter than the typical travel time of the solar-wind from Sun to the Earth: two to four days.

## Output Data at JSOC

The MHD data are available, along with the other HMI products, at the website jsoc.stanford.edu/ajax/lookdata.html. In most cases, users have to provide three pieces of information to specify a particular data: the series name, the primary keyword value, and the segment. The series name is an identifier, at the topmost of the database-tree structure, to specify the type of data. The primary keyword is to specify a record (a minimum set of data to be physically or observationally meaningful), typically a time. The segment, the lowermost layer of the database, specifies the name of a physical or observational variable.

### Series Name, Record, Name of Segment, and Keyword

Table 3 tabulates the series names of the MHD products, (A), (B), and (C), together with the input magnetic-field map.

**Table 3:** Summary of the MHD runs and the input HMI magnetogram map data.

Label	Output series name	Input series name	Cadence
a	hmi.MHDcorona	hmi.Synoptic_Mr_720s	27 or 28 days (1 Carrington rot.)
b	hmi.MHDcorona_daily	hmi.Mrdailysynframe_720s	1 day
c	hmi.MHDcorona_daily_nrt	hmi.Mrdailysynframe_ 720s_nrt	1 day

The primary keyword, an index of time, of the definitive once-per-CR simulation data is the Carrington rotation number. For example, the MHD data for CR 2140 are specified as hmi.MHDcorona[2140].

As for the daily MHD data, each data record is specified with the time T_REC of the magnetic-field data map that is usually denoted in TAI time format, YYYY.MM.DD_HH.MM.SS. On most days, the daily updated magnetic-field maps are generated and time-stamped around 1200 UT. Thus, the hour and minute of the daily input magnetic-field and the MHD product, HH.MM, are 12:00, or neighboring 12-minute slots, 11:48 or 12:12 less frequently. The two-digit second [SS] is always zero.

The names of segments in the MHD product are notation of physical variables, *i.e.*N, T, Vr, Vt, Vp, Br, Bt, and Bp (plasma number density, temperature, the radial, latitudinal, and longitudinal component of plasma velocity, and the three components of the magnetic field, respectively). Table 4 lists the segments, the physical variables, and the units used in storing the data values.

**Table 4:** Segments of coronal MHD data cube.

Segment name	Description	Notation	Unit
N	Number density	*N*	10^8^ cm^−3^
T	Temperature	*T*	10^6^ K^∘^
Vr	Radial component of plasma flow speed	*V*_*r*_	km s^−1^
Vt	Latitudinal component of plasma flow speed	*V*_*θ*_	km s^−1^
Vp	Longitudinal component of plasma flow speed	*V*_*ϕ*_	km s^−1^
Br	Radial component of magnetic field	*B*_*r*_	Gauss
Bt	Latitudinal component of magnetic field	*B*_*θ*_	Gauss
Bp	Longitudinal component of magnetic field	*B*_*ϕ*_	Gauss

### Keywords

In the JSOC database context, a large set of keywords collects information relating to the observation, calibration, or other post-processes, such as the time of observation, telescope setting and state of the spacecraft, and code version number. Because the FITS file generated at the JSOC website contains most of the JSOC keywords in the header field, it is in most cases appropriate to regard the JSOC keywords as the FITS header information.

Many keywords of the MHD products are copied from the input magnetic-field data set, and some of them are the same values and others may be updated accordingly. There are several keywords newly added to describe the basic nature of the MHD simulation. Tables 5 and 6 summarize the most relevant keywords, those succeeded from the input magnetic-field data, and those updated by the MHD module. A complete list of keywords can be found at the JSOC webpage: jsoc.stanford.edu/ajax/lookdata.html.

**Table 5:** Selected keywords with the same value as those of the input data. In the rightmost three columns, “x” indicates that the output MHD data by (A) moderate-resolution once-per-CR simulation using definitive synoptic map, (B) low-resolution simulation using daily definitive synchronic map, or (C) low-resolution simulation using daily NRT synchronic map has the keyword. The upper case “X” indicates that the keyword is the prime keyword used to identify the data record: The MHD data series and the magnetogram map series share the same prime keyword.

Name	Type, value, [unit]	Description	(A)	(B)	(C)
T_REC	TAI time format	Time of Record		X	X
CAR_ROT	4-digit integer	Carr. Rot. Num. of obs.	X	x	x
CARRTIME	[degree]	Carrington time in float	x	x	x
MAP_DATE	ISO 8601 format UTC	date of input map creation	x	x	x
MAP_CVER	string	Code version info. of synoptic/synchronic map module	x	x	x
MAP_BLDV	string	Build number of synoptic map module	x	x	x
CADENCE	86,400	Cadence of input data [seconds]	x		
	360	[degrees]		x	x
T_OBS	TAI time format	nominal time of observation		x	x
T_REC_epoch	TAI time format	Time of origin		x	x
T_REC_step	720	ts_eq step		x	x
T_REC_unit	“secs”	ts_eq unit		x	x
T_START	TAI time format	Carrington Rotation Start Time of input map	x		
T_STOP	TAI time format	Carrington Rotation Stop Time of input map	x		
T_ROT	TAI time format	Carrington Rotation Middle Time of input map	x		
B0_ROT	[degree]	B-zero angle [*B*_0_] of map center	x		
B0_FRST	[degree]	B-zero angle [*B*_0_] at T_START	x		
B0_LAST	[degree]	B-zero angle [*B*_0_] at T_LAST	x		
LON_FRST	[degree]	First Carrington Time of input global map	x	x	x
LON_LAST	[degree]	Last Carrington Time of input global map	x	x	x
ORIGIN	“SDO/JSOC-SDP”	ORIGIN: location where file was made	x	x	x
TELESCOP	“SDO/HMI”	For HMI: SDO/HMI	x	x	x
INSTRUME	“HMI_SIDE1”	For HMI: HMI_{SIDE1, FRONT2, COMBINED}	x	x	x
CALVER64	hex	Calibration Version	x	x	x
COMMENT		Supplemental Comments	x	x	x

**Table 6:** JSOC keywords of the MHD data: new or updated by the MHD modules. Asterisks (*) are placed next to the keywords newly added by the MHD modules. Keywords without the asterisks in this table are of the same name as those of the input magnetic-field maps but given different values.

Name	Type, value, [unit]	Description	(A)	(B)	(C)
MHD_VER1 *	string	version of JSOC interface code	x	x	x
MHD_VER2 *	string	version of MHD code	x	x	x
MHD_SET1 *	string	setting in coronal MHD model	x	x	x
MHD_SET2 *	string	setting in coronal MHD model	x	x	x
MHD_SET3 *	string	setting in coronal MHD model (for future use)	x	x	x
MHDIBMAG *	string	initial and boundary mag. setting	x	x	x
MHDMGIDX *	integer	index of initial mag. setting	x	x	x
MHDMODEL *	string	supplemental comment about model	x	x	x
INPUTMAP *	string	input magnetic global map identifier	x	x	x
DATE	ISO 8601 format UTC	time of processing	x	x	x
CRPIX1	72.5	location of the image center	x		
	36.5			x	x
CRPIX2	36.5	location of the image center	x		
	18.5			x	x
CRPIX3 *	40.5	location of the image center	x	x	x
CTYPE1	“CARL-CAR”	Carrington time	x	x	x
CTYPE2	“CRLT-CAR”	Heliographic latitude	x	x	x
CTYPE3 *	“HECR”	Radial distance from sun center	x	x	x
CRVAL1	[degree]	Carrington time at center of the data	x	x	x
CRVAL2	0.0	Latitude at center of the data	x	x	x
CRVAL3 *	3.0	Heliocentric distance at center of the data	x	x	x
CDELT1	−2.5	image scale in the 1st direction (Carrington time)	x		
	−5.0			x	x
CDELT2	2.5	image scale in the 2nd direction (latitude)	x		
	2.5			x	x
CDELT3 *	0.05	image scale in the 3rd direction (radius)	x	x	x
CUNIT1	“degree”	Unit of scale in the 1st direction	x	x	x
CUNIT2	“degree”	Unit of scale in the 2nd direction	x	x	x
CUNIT3 *	“solRad”	Unit of scale in the 3rd direction	x	x	x
WCSNAME	“3D-SPHERICAL”	WCS system name	x	x	x

### Data Grid System

Our MHD model employs a non-uniform grid size along the radius. For various reasons, the number of grid points in latitude and longitude have to be a power of two (2^5^ for the low-resolution and 2^6^ for the medium-resolution simulation). This grid size was chosen after taking into account the steep gradient of the density and temperature near the solar surface and some computational efficiency. This simulation grid system may not be convenient for general use, so we reorganize the data.

The MHD data cubes available in the JSOC database segments are linear-interpolated spatially into equi-distance for the radial direction and equi-angle for the latitudinal and longitudinal directions. We choose a right-hand system where the first position address runs for the longitudinal direction (from East to West in the Carrington longitude), the second for the latitude (from South to North), and the third one for the radius. In this format, a three-dimensional address [*i*,*j*,*k*] positions a grid point [*ϕ*_i_,*θ*_j_,*r*_k_],

6 $$ \phi_\mathrm{i} = (i+1/2)\Delta\phi, \qquad \theta_\mathrm{j}=-90+(j+1/2) \Delta\theta, \qquad r_\mathrm{k} =1.0+(k+1/2)\Delta r. $$

Note that here each address index starts from zero.

We note that this data coordinate system has an opposite sense in latitude to those of the MHD code where the (co-)latitude is oriented from North to South. To be fully self-consistent in the production data, the segments, Vt (for *V*_*θ*_) and Bt (for *B*_*θ*_), are set in a way such that positive values stand for the component oriented northward.

The longitude of the input synoptic/synchronic maps in the JSOC database is defined as the Carrington time, which runs from West to East, in an opposite sense to the ordinary definition of longitude. As the downstream product of the magnetic-field map, the MHD production data have to use the same definition. To keep the right-hand system, on the other hand, we wish to keep the address for the longitudinal direction in the cubic data. To satisfy all requirements, we define the integer address running from East to West in the same manner as the ordinary longitude in spherical coordinates, while its incremental step is defined to be a negative value. In this definition, the Carrington time can be calculated as

7 $$ \phi_{\mathrm{CR},i} = 360 + (i+1/2)\Delta\phi_{\mathrm{CR}}. $$

The angular increment steps in the longitudinal and latitudinal directions [Δ*ϕ*_CR_ and Δ*θ*] are recorded as a value of the keywords CDELT1 and CDELT2. As summarized in Table 5, the values are fixed in the same series, and the values are multiples of 2.5 degrees, *i.e.* (Δ*ϕ*_CR_,Δ*θ*) are (−2.5,2.5) for the medium-resolution simulation (A), and (−5.0,5.0) for the daily low-resolution simulations (B) and (C).

**Figure 4:**
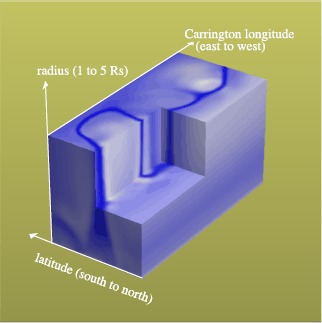
A cut-away view of cubic, three-dimensional synoptic data. The plasma density [*N*] normalized with an average value at each height (heliocentric distance) obtained with simulation (A) for CR 2145 is shown. On the top face (at 5.0 R_⊙_), the high-density streamer belt is visible. On the left face (at zero degrees longitude), the cross-section of a streamer is visible near the heliographic Equator, and another high-density region corresponding to the polar crown closed-field region can be seen at northern high latitude.

Figure 4 shows a cut-away view of the coronal density normalized with its average at each height as an example for CR 2145. For this period, a highest-density belt is seen in the topmost layer at 5.0 solar radii, which corresponds to the base of the HCS and the topmost part of coronal streamers. The evolution of the streamer with respect to height can be seen on the left face (the meridional plane at longitude 0 degree). Below about three solar radii, the field lines are closed, and the outermost layer of the closed-field structure has a higher density than the outer part of a coronal hole and the inner part of the streamer. Above about three solar radii, many of the magnetic fields are open to interplanetary space, and the plasma is no longer confined. Still, the speed is slower and the density is higher than in the surroundings, and the high-density regions can reach the interplanetary space.

On the left, another short-height closed-field region can be seen in the northern hemisphere (left). This high-density structure near the Pole corresponds to the surroundings of the polar crown. The structure forms a dome over the (northern) Pole whose magnetic polarity is opposite to that of the surrounding high latitudes. This structure may be somewhat common in the maximum phase of solar activity; the reversal of the interplanetary polar field takes place before the highest-latitude solar-surface magnetic field reverses its polarity.

## Data Demonstrations

The MHD modules provide the plasma variables (density and temperature) as well as the magnetic field in the solar corona. Because the derived structures are a consequence of the nonlinear interaction between the plasma and magnetic field, the derived magnetic-field structures specifically at the upper corona are different from those obtained through the PFSS model. This section exhibits the coronal plasma density, shapes of open-field region, and the magnetic field escaping to interplanetary space, derived from the daily and once-per-CR simulations. We chose a period from late 2010 to early 2011.

### Coronal Density

First, we examine the plasma density in the corona, which is one of the derivative products from the photospheric HMI observables. The derived density is generally highest at the base of the HCS near the top of coronal streamers and is noticeably higher at the unipolar boundary layers than in the open-field corona holes. Therefore, the computed density is a good proxy of the global magnetic field structure.

**Figure 5:**
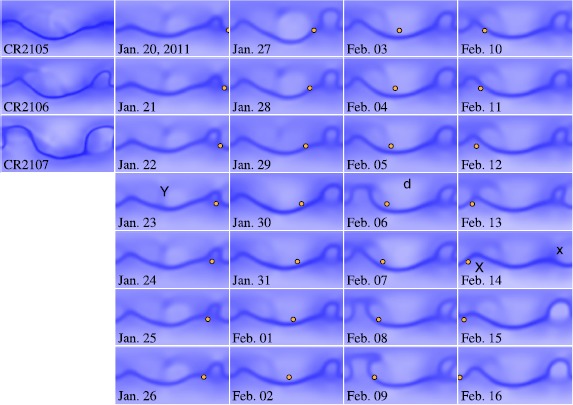
Density in the topmost layer of the data cube at about 5.0 R_⊙_ from North to South Pole in latitude and from 0 to 360 degrees Carrington longitude. The white-blue color table spans the density range from 0.9×10^5^ (white) to 1.4×10^5^ cm^−3^ (blue). The highest density (dark blue) corresponds to the lower-speed current sheet or the uppermost part of a stagnant coronal streamer, and the brighter blue corresponds to open-field regions with lower density and faster outward flow. In the leftmost column, the density maps derived from the medium-resolution simulation (A) for three consecutive Carrington rotation numbers from 2105 to 2107 are shown for reference. The second to fifth column show the density maps obtained through the low-resolution daily simulation using definitive daily maps (B) from 2011 Jan 20 (corresponding the start date of CR 2106) to 16 February 2011 (the last day of CR 2106). An orange circle is placed at the approximate position of Earth. The mark “Y” on the map of 23 January indicates where three unipolar boundary layers are merging, and the “d” on 6 February indicates the disappearance of the unipolar boundary layer. The mark “x” indicates the approximate position of the two Active Regions, NOAA 11147 and 11161, which appeared at almost the same heliographic position in two consecutive solar rotation periods. The mark “X” on 14 February is placed at the position of NOAA 11158.

Figure 5 shows the density structures at 5 R_⊙_, the topmost data layer in the production data cube, over 28 days from 20 January to 16 February, derived from the daily simulation (B). The same-format plots of the higher-resolution once-per-CR simulation (A) from 2105 to 2107 are shown in the leftmost column (the central time of CR 2106 falls on 3 February; the approximate center of the 28-day time span), for reference. Overall, the highest-density belt obtained from the daily simulations follows that from the once-per-CR simulation, evolving gradually on the time scale of a few weeks to a month. At the same time, however, we also find that variations of the global corona can be sudden and take place in a day or two. The merit of the daily simulation is that it can catch such sudden changes in the global corona that might be missed when we only conduct once-per-CR simulations.

We identify two factors that can cause sudden changes in the daily simulation results: The first is the change in unbalanced flux in the global map, and the other is due to local, compact sudden changes such as emergence of magnetic flux forming solar active regions. In the current version of the MHD module, we simply offset the apparent unbalanced flux in a synoptic frame by subtracting the total surface integration of *B*_*r*_ divided by the area $[4\pi~\mbox{R}_{\odot}^{2}]$ evenly everywhere on the map so that the resulting map will have a fully balanced magnetic field. We recognize the possibility that this offsetting can result in changes of the position of the magnetic inversion line (MIL) on the global scale and thus the global structure of the simulated solar corona. Such offsets are often due to an active regions rotating into or out of the synoptic frame. However, the effects of the offsetting may appear on the global scale in the simulation results, such as by displacement of the position of the high-density belt in a specific direction. On the other hand, most of the local variations in the results from the PFSS and MHD can be attributed to local, sudden changes of the solar photospheric magnetic field, because the sensitivity of the solution of the PFSS (or Laplace equation) decreases very quickly with distance. Therefore the solutions of the MHD simulation using the PFSS solution for the initial value have the same tendency.

We can find belts with moderately high density (*i.e.* lower than those at the base of HCS but higher than the surroundings ordinary coronal holes) North of the HCS highest-density belt. These regions correspond to unipolar boundary layers (UBL) or pseudo-streamers where the plasma and magnetic flux from two coronal holes with the same polarity interact to form a region of moderately high density and slow plasma flow. In the series of daily plots in Figure 5, a Y-shaped UBL is found in the northern hemisphere on the first several days (marked “Y” in the 23 January box), then, around 30 January, the western part of this Y-shaped structure disappeared (marked with “d” in the 6 February box). The UBL is a consequence of the balance between the plasma and magnetic field: If the magnetic field is weaker, then two closed-field streamers with a coronal hole between them will be formed. We need to be careful when we evaluate whether simulated structures fully match the real ones, although we emphasize that the changes found in the daily simulation can signal a possible large-scale reconfiguration of the coronal magnetic field.

In the rightmost column, we can see clear changes in a northward bulge of the high-density belt (marked with “x” in one box). The bulge at about 300 degrees Carrington longitude gradually expanded and then retained its size until 14 February, when the bulge structure mostly disappeared, and then it regained its size on the next day. The daily updated maps started to include the X-flare Active Region 11158 on February 13 (marked with “X” in one box) and no significant changes are found in Figure 5, while two other Active Regions, NOAA 11147 (seen at about 340 degrees Carrington longitude and 20 degrees North heliographic latitude in the previous solar rotation period) and NOAA 11161 (mostly at the same heliographic location; about 330 degrees of longitude and 15 degrees North), were rotating into the longitudinal range of the daily updated maps on 15 February. Therefore, the evolution of the simulated bulge structure around 15 February is mainly due to the magnetic field at the active regions with a large separation angle (about 60 degrees), NOAA 11147 and 11161 (in the northern hemisphere), and the NOAA X-flare 11158 (locating at about 30 degrees Carrington longitude and 20 degrees South). With only once-per-CR simulations, we do not register these possible day-by-day variations in the global solar corona and solar wind.

### Comparisons with AIA: Shape of Open-Field Coronal Holes

A coronal hole is a good proxy of an open-field region in the solar corona. From the simulated data, the open field can be calculated by tracing the field line; therefore comparing simulated open field regions and dark regions in coronal observation is a good benchmark for checking the simulation data.

**Figure 6:**
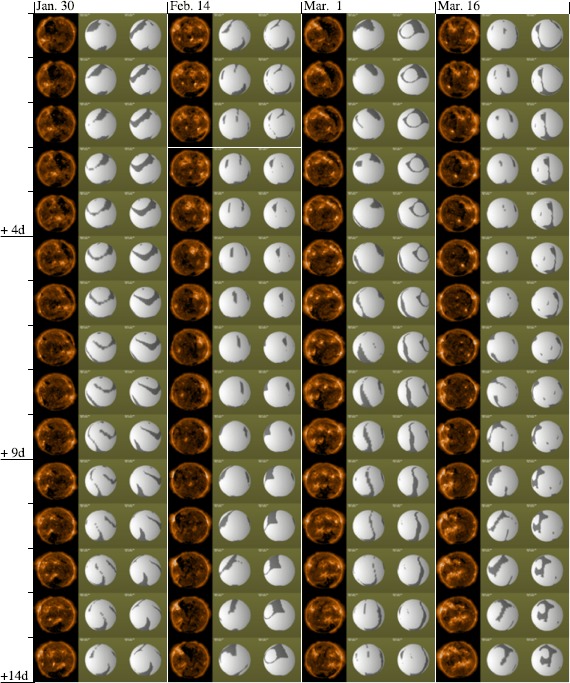
Comparisons of shapes of the simulated open-field regions with the AIA 193 Å  observation images. In each column, from left to right, the AIA image, the daily low-resolution MHD simulation (B), and the definitive simulation results from once-per-CR simulation (A) are drawn. The brown for AIA image data is adjusted so that low-intensity parts are darker and the coronal holes are clearly visible. The bases of the open-field (closed-field) regions of the simulated corona are colored with darker (brighter) gray. The viewpoints in the simulation plots are set at the position of Earth at 1200 UT each day. Horizontal white lines are placed at the top of the box for the first day of Carrington Rotation 2107. Carrington Rotation 2108 started early on 16 March 2011.

Figure 6 shows the AIA 193 Å  image data taken around 12 UT each day, the solar-surface base of the open-field region derived from the low-resolution simulations (B) using the daily updated synchronic map, and the medium-resolution simulations (A) using the once-per-CR medium-resolution simulation over 60 days from 30 January to 30 March 2011. The coronal bases of the open-field region for the daily simulation (B) are viewed from the position of Earth at the time of the daily observation. Each plot for the simulation (A) is made by showing the simulation data for the corresponding Carrington rotation period as viewed from the Carrington longitude and heliographic latitude [*B*_0_] of Earth at Noon UT on each date. The horizontal white lines show the dates at which the Carrington rotation number increases and thus when the input data for the simulation (A) changes.

Overall, reasonable agreements with the AIA data are found in the locations and shapes in both daily simulation (B) and the once-per-CR simulations (A), although discrepancies are also recorded. To achieve the best agreement, we probably need to tune parameters such as the plasma density and temperature and the polytropic index more finely, although such fine-tuning is not within the scope of the simulation embedded into the HMI data pipeline.

A noticeable disagreement between the daily simulation (B) and AIA image data is found around 15 February: the numbers of open-field regions in the Earth-facing side of the northern hemisphere simulated with the daily simulation (B) were 2, 1, and 2 on 13, 14, and 15 February, respectively, while the coronal holes in the AIA image data were rather stable and did not show such changes. This can be attributed to the update window in the daily updated map production step, where the updated region is limited to the 60-degree window around the central meridian. With the fixed update window size, it is possible that only part of the solar active region is included in the updated map, which can cause a substantial imbalance of the magnetic-field polarity, which can affect the results of the PFSS and MHD models.

### Comparisons with Near-Earth *in-situ* Data

The near-Earth *in-situ* data are a good reference for checking the model outputs. For comparison with the 1-AU *in-situ* data, the radial component of the magnetic field [*B*_*r*_] is a suitable parameter because this is a conservative physical quantity. Moreover, the sign of *B*_*r*_ in interplanetary space is the combined consequences of the photospheric field and coronal conditions that we model. Because the solar wind typically takes about four days to travel from the Sun to Earth, it is reasonable that the simulated value of the solar wind near the Sun at the heliographic position of Earth predicts the value at Earth four days later quite well. In the same way, the state of the solar wind at Earth at an instant is linked to the near-Sun value at a Carrington longitude about 53 degrees West of the current Sun–Earth direction.

**Figure 7:**
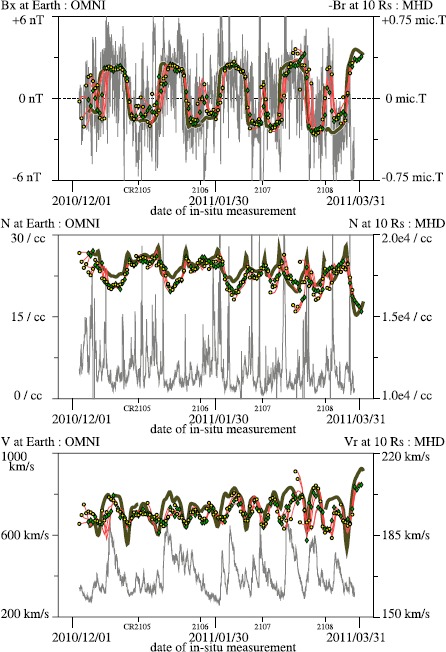
Comparisons of the Sun–Earth component of the interplanetary magnetic field, plasma number density, and flow speed, from 1 December 2010 to 31 March 2011. Gray lines show the hourly averaged *in-situ* data, from OMNIWeb database. The green-filled diamonds (yellow-filled circles) indicate the values obtained from the daily MHD simulation (B) sampled at the heliographic position of the Earth at 1200 UT, four days after (the same date as) the corresponding *in-situ* measurement, at 10 R_⊙_. The red lines connecting the two circles show the values at the 1200 UT of the three days in between in the same daily simulation data. Dark-brown lines show the simulated values from the medium-resolution simulation (A), prepared in the same manner as the green-filled diamond marks. Short vertical lines with Carrington rotation numbers are placed at the dates four days earlier than the start date of each rotation period.

Here, we use the simulated *B*_*r*_ at the heliocentric distance of ten R_⊙_ to compare it with the *in-situ* data. The top plot of Figure 7 shows the Sun–Earth component of the interplanetary magnetic field (IMF), that is *B*_*x*_ from the OMNI database and the corresponding simulation parameter [−*B*_*r*_]. In the plot, the hourly averaged *B*_*x*_ in the OMNI database is drawn with thin gray lines. The green-filled diamonds and yellow-filled circles show the value of simulated −*B*_*r*_. The green diamond is placed in the plot at the position four days after the date for which the daily simulation (B) was computed. The yellow circle is placed at the date of the *in-situ* measurement and the input magnetic-field map, but the value is sampled at about 53 degrees West of the Sun–Earth direction. A red curve connects the circle and diamond and shows the values on the three days in between. A green-filled diamond and red line show, in this way, a prediction for four days. Thick dark brown curves show the same quantity as the green-filled diamond derived from the once-per-CR medium-resolution simulations (A). Overall, the simulation data agree well with the *in-situ* near-Earth measurement.

The once-per-CR simulation (A) is not very good at reproducing the data near 0 or 360 degrees Carrington longitude well because of the discontinuity of the input solar photospheric magnetic-field maps. On the other hand, the daily simulation (B) gives better results around the start or end date of a Carrington rotation period. We will be able to improve the results of the higher-resolution simulation by conducting it more frequently, for example, twice per CR rotation, using an extra synoptic map from 180 degrees Carrington longitude to 180 degrees of the next rotation period.

The yellow-filled circle points, which represent the now-cast, often trace some peak values of the *in-situ* data better than the green-filled diamond points that are derived from simulations using the magnetic-field data four days earlier. However, overall, both agree moderately well with the *in-situ* data, and here we refrain from attempting to judge which simulation data value is better than the other. Instead, we point out here that the solar wind can often be affected by the condition of the photospheric magnetic field at a distant solar surface region, for example about 90 degrees East from its origin. In the daily simulation (B), the solar-surface region 37 degrees East from the Sun–Earth direction (90 degrees East from the assumed origin) can be included in the daily updated synoptic map made for the day of the *in-situ* measurement but has not been included in the map made four days earlier.

In the figure, the scale for the simulated −*B*_*r*_, ± 0.75 μ (micro) Tesla corresponds to 6 nT, if it decreases with 1/*r*^2^, 1.62 (=750/21.5^2^) nT at 1 AU, which is about a quarter of the scale used for the *in-situ* data. The deficiency of the magnetic-field strength derived from the coronal models, the so-called missing flux, is an interesting and unsolved topic in the field of the solar wind and interplanetary dynamics. Because there is no consensus about the causes of the mismatch, we do not apply any adjustment, for example multiplying values of *B*_*r*_ by a factor of four, to the input magnetic-field map or simulated data. As a data-pipeline product, the MHD simulation data and the magnetic-field maps are published as is.

The middle and bottom panels of Figure 7 compare the simulated plasma number density and flow speed at ten solar radii with the OMNI data. In these plots, different offsets and scales are applied to enhance faint contrasts of these plasma quantities that were simulated with polytropic assumption. In the middle plot, many of the maxima and minima of the simulated density coincide with those of the 1-AU measurements. The correlation coefficients are reasonably good: about 0.35. Although the contrast of the simulated density is much fainter than in the *in-situ* measurement data, a key feature of the solar corona, the enhancements of the simulated density at the closed-field streamer or pseudo-streamer, can be reproduced reasonably well. In the bottom plot, the flow speed greater than 500 km s^−1^ in the *in-situ* data and the maxima of the simulated plasma flow speed coincide well, although the contrast of the simulated flow speed is fainter. The simulated higher-speed plasma flow originates from the open-field coronal hole. Therefore, these reasonable agreements in plasma parameters imply that the simulated coronal magnetic field, both open- and closed-field structures, is reasonable overall.

We note that the mass flux of the simulated solar wind tends to be much higher than that measured at Earth. In the four-month period shown in Figure 7, the mass flux of the simulated solar wind near the solar Equator at ten solar radii is about 3.5×10^6^ km s^−1^ cm^−3^ and the average of the mass flux measured near the Earth is about 2×10^3^ km s^−1^ cm^−3^. Hence the simulated mass flux is about four times as high as in reality ((3.5×10^6^)/(2×10^3^)÷(215/10)^2^≈3.79). The surplus can be reduced by applying the boundary conditions that are capable of limiting the boundary mass flux (Hayashi, 2005). As a standard data-pipeline product, however, the MHD module in the JSOC uses a simple boundary condition without any mass-flux limits on the solar coronal base.

## Summary and Discussion

This article described the MHD simulations that are part of the JSOC data pipeline and the specifications of the output data at the JSOC database interface.

Three types of the MHD simulations, labeled (A) to (C) in this article, are conducted regularly in the HMI data pipeline. A medium-resolution simulation conducted once per Carrington rotation period (labeled (A)) and two versions of the daily MHD simulation: One uses the NRT data, the other the definitive, calibrated synchronic map data (labeled (B)). The daily NRT simulations labeled (C) are primarily intended to provide the community with quick-look data to help assess the current state of the plasma quantities of the solar corona, and the definitive daily simulations provide reliable solutions and supplement the NRT simulations. For this purpose, we made some compromises in the simulation setting to minimize the computational resources required and accomplish timely productions regularly.

The settings that we chose for making the global magnetic-field map and the MHD simulation of the corona are optimized overall in the context of the HMI data pipeline to provide the simulation data in a timely manner. The simulation results are compared with the AIA image data and the OMNI near-Earth *in-situ* measurement data, and reasonably good agreements are obtained, meaning that the selected parameters are suitable.

There are three options and functionalities that we did not use in the current version, but will include in the future:

The first one is to correct for the polar field when constructing the photospheric *B*_*r*_ map. We have developed a method and tested it with SOHO/MDI data (Sun *et al.*, 2011) that uses temporal and spatial polynomial interpolation to fill the missing data at the Poles. This polar-field correction needs observational data over three years to yield reliable temporal interpolation and one year of data to validate, which was fulfilled by the end of 2014. The JSOC data pipeline started making synoptic maps and synchronic maps using HMI vector data. Using the vector-based *B*_*r*_ maps for modeling the solar corona (*e.g.* Hayashi *et al.*, 2013) is an additional option to improve the input maps.

The second option is in treating the active regions near the longitudinal edges of the whole-surface maps. In making daily synchronic frames and once-per-CR synoptic maps, it is often the case that only part of the sunspot group is included in the whole-surface map, which can result in a substantial imbalance of the magnetic flux and hence displacement of the magnetic neutral lines in the offset map. Thus, making an improved global map needs the algorithm to recognize magnetically active regions on the solar surface. It is a reasonable expectation that the HMI Active Region Patches (HARP) module (see Bobra *et al.*, 2014 and Turmon *et al.*, 2015), already implemented in the HMI vector magnetic data production for automatically identifying magnetically active regions, can improve global map production (Hayashi *et al.*, 2012; Hoeksema *et al.*, 2014). These two features, if implemented, will help expand the ways in which whole-surface maps can be used in the models for the global corona such as MHD and PFSS.

The third point is to apply time-dependent boundary conditions to the MHD simulation. Because the solar corona is a sub-Alfvénic system, the state of the solar corona at an instant is influenced by not only the boundary values at that instant, but also by values in the past. Therefore, it is desirable for the MHD model to be capable of handling time-dependent boundary data. The global variation of the magnetic field, caused by the differential longitudinal flow and the meridional flow, can produce the formation and evolution of large-scale twisted structures that can erupt into interplanetary space (*e.g.* Linker *et al.*, 2001; Yeates, Mackay, and van Ballegooijen, 2008; Yeates and Mackay, 2012; Yang *et al.*, 2012; Feng *et al.*, 2012; Hayashi, 2013). A similar strategy can be applied to interplanetary space (*e.g.* Hayashi, 2012) and active regions (*e.g.* Cheung and DeRosa, 2012; Jiang *et al.*, 2013; Inoue *et al.*, 2013). Because implementing the boundary treatment used in these models, at least ours (Hayashi, 2012; Hayashi, 2013), is not computationally expensive, it will not be a hard task to develop a new version of the MHD modules for the HMI data pipeline. If successful, the new module will be able to provide MHD solutions of the global solar corona that evolves seamlessly in time.

The codes for these three items were already used in other modules in the HMI pipeline or by the HMI team. The whole-surface magnetic-field map and the MHD results with the new choices will be published as a new series of data in the JSOC database, and the current version will continue to be generated.
